# Q&A: using Patch-seq to profile single cells

**DOI:** 10.1186/s12915-017-0396-0

**Published:** 2017-07-06

**Authors:** Cathryn R. Cadwell, Rickard Sandberg, Xiaolong Jiang, Andreas S. Tolias

**Affiliations:** 1 Department of Neuroscience, Baylor College of Medicine, One Baylor Plaza, Houston, Texas 77030 USA; 2 Ludwig Institute for Cancer Research, Stockholm, Sweden; 3 Department of Cell and Molecular Biology, Karolinska Institutet, Nobels väg 3, 171 65 Solna, Stockholm, Sweden; 4 Jan and Dan Duncan Neurological Research Institute at Texas Children’s Hospital, 1250 Moursund Street, Suite 1025.18, Houston, Texas 77030 USA; 5 Department of Electrical and Computer Engineering, Rice University, Houston, Texas USA

## Abstract

Individual neurons vary widely in terms of their gene expression, morphology, and electrophysiological properties. While many techniques exist to study single-cell variability along one or two of these dimensions, very few techniques can assess all three features for a single cell. We recently developed Patch-seq, which combines whole-cell patch clamp recording with single-cell RNA-sequencing and immunohistochemistry to comprehensively profile the transcriptomic, morphologic, and physiologic features of individual neurons. Patch-seq can be broadly applied to characterize cell types in complex tissues such as the nervous system, and to study the transcriptional signatures underlying the multidimensional phenotypes of single cells.

## What is Patch-seq?

**Fig. 1.**
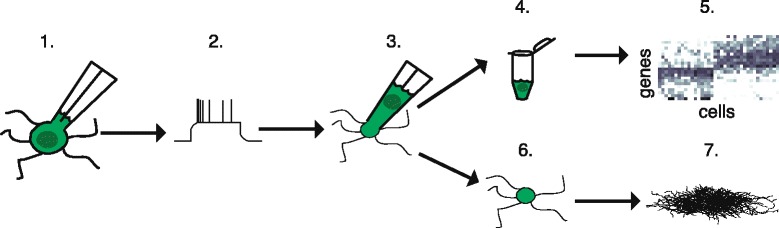
Overview of Patch-seq technique. Access to the intracellular compartment of a single neuron is gained by whole-cell patch clamp (step 1) and the electrical properties of the cell, such as its firing pattern in response to depolarizing current injection, are recorded (step 2). The intracellular contents are aspirated into the patch pipette (step 3) and collected in a PCR tube (step 4) for downstream RNA-sequencing (step 5). The tissue slice, which retains the collapsed cell body and fine processes of the cell (step 6), is subjected to immunohistochemical staining to visualize the complex morphology of the cell (step 7). Adapted by permission from Macmillan Publishers Ltd: *Nature Biotechnology* [1], copyright (2016)

The term ‘Patch-seq’ refers to the combined application of whole-cell patch clamp recording and single-cell RNA-sequencing (scRNA-seq) to individual cells. In parallel with another group led by Sten Linnarsson and Tibor Harkany, we recently developed the Patch-seq technique and applied it to study neurons in the mouse cortex [1, 2]. While there are several differences between the two protocols (see below), the basic approach is the same: after a cell is patched and its intrinsic electrophysiological properties are recorded, the intracellular contents are aspirated into the patch pipette and used for scRNA-seq (Fig. 1). In contrast to other scRNA-seq methods, which utilize dissociated cells [3–5], Patch-seq can be applied to study single cells in situ in live tissue slices [1, 2] or even intact animals [1], making information about the anatomical position, morphological structure, electrical properties, connectivity, and function of the cell within the local circuit simultaneously accessible. The multimodal datasets generated using Patch-seq can enable scientists to examine the relationship between genome-wide expression patterns and phenotype with unprecedented single-cell resolution.

## What are the main applications of Patch-seq?

Patch-seq can be applied to answer a multitude of scientific questions that require correlating gene expression with physiology and/or morphology at the level of single cells. For example, Patch-seq provides an unbiased strategy to characterize and classify cell types by integrating information about each cell’s morphology, physiology, and gene expression into a common framework. Patch-seq can also be used as a complementary method to ‘annotate’ cell type classification based primarily on scRNA-seq of dissociated neurons; in other words, to link molecular cell types with their corresponding morphology and physiology. The generation of a comprehensive cell type atlas with genome-wide expression data may lay the foundation for a more principled understanding of neuropsychiatric diseases by identifying the specific functional cell types that express disease-associated genes. In addition to cell type studies, we envision that Patch-seq can be broadly applied, for example, to study the transcriptional changes that occur within a single cell during plasticity, or combined with transgenic, viral, and optogenetic techniques to explore the transcriptional signatures of neurons with a specific developmental lineage, neurons that project to a particular brain region, or neurons that receive input from a common brain region. By combining Patch-seq with multiple simultaneous whole-cell recording techniques to study connectivity [6] we may be able to decipher the molecular mechanisms that underlie cell type-specific connectivity. Patch-seq could also be used to profile cell types of other complex organs outside the nervous system. In summary, we believe that Patch-seq is a powerful tool that can enhance many research programs and permit new avenues of investigation into the molecular underpinnings of cellular diversity.

## What differences are there between Patch-seq protocols?

There are currently two published protocols for Patch-seq, our own [1] and that of Fuzik et al. [2]. There are several important modifications to the standard patch clamp procedure (Table 1) that both protocols share, including strict RNase-free preparation of solutions and equipment used for collecting single-cell RNA samples, the use of large patch pipette tip sizes (that produce lower resistance than typically used for patching), use of a small volume of internal solution in the patch pipette, and the addition of ethylene glycol-bis (β-aminoethyl ether)-N,N,N′,N′-tetraacetic acid (EGTA) to the internal solution [7]. The major differences between the two protocols lie in the composition of the internal solution and the sequencing method used. In addition to EGTA, our internal solution also includes glycogen and RNase inhibitor. We included glycogen because of previous reports suggesting that it improves RNA yield [8, 9] and we found in pilot studies that the addition of RNase inhibitor increased cDNA yield approximately threefold [1]. The protocol described by Fuzik et al. did not include either glycogen or RNase inhibitor in the internal solution, but did report the use of depolarizing current steps prior to aspiration of the cell contents to facilitate entry of RNA into the pipette. While we have not observed an increase in cDNA yield following depolarizing current injection, in our experience this may help to diffuse biocytin throughout the cell and improve morphological recovery (unpublished observations). Our cDNA amplification and library construction protocol is based on the Smart-seq2 method for sequencing full-length cDNA [10–12], while the protocol developed by Fuzik et al. uses a unique molecular identifier (UMI)-based single-cell tagged reverse transcription (STRT) protocol to reduce PCR bias at the cost of sequencing only the 5′ end of each cDNA molecule [3, 13].

**Table 1:** Modifications to standard patch-clamp procedure for Patch-seq

Modification	Standard patch clamp	Patch-seq [1]	Patch-seq [2]	Purpose
RNase-free precautions	−	✔	✔	Prevent sample degradation by exogenous RNase
Large patch pipettes	−	✔	✔	Easier to aspirate cell contents into pipette
Small volume of internal solution	−	✔	✔	Prevent interference with downstream reactions and loss of sample RNA
EGTA in internal solution	−	✔	✔	Scavenge free calcium to reduce activity of endogenous RNase
Glycogen in internal solution	−	✔	−	RNA carrier
RNase inhibitor in internal solution	−	✔	−	Reduce activity of endogenous RNase
Depolarizing current steps	+⁄−	+⁄−	✔	May facilitate diffusion of biocytin into cell and RNA into pipette
Sequencing method	−	Smart-seq2-based	STRT-based	Full-length gene coverage [1] or reduce PCR bias [2]

## Can morphology of sequenced cells be directly assessed?

**Fig. 2.**
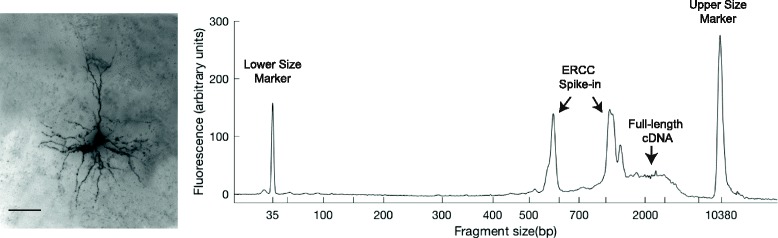
Combined Patch-seq and morphological recovery. Immunohistochemical staining (*left*, *scale bar* 50 μm) and full-length amplified cDNA Bioanalyzer profile (*right*) from a layer 2/3 pyramidal neuron. ERCC spike-in RNA was used as a positive control in this experiment and gives rise to the distinct peaks at ~600 bp and ~1100 bp in the Bioanalyzer profile

In our original Patch-seq protocol [1], morphology was inferred based on the cell’s electrophysiological properties, but this approach may not be possible for cell types that have very similar electrophysiological properties. Thus, direct morphological recovery of cells following Patch-seq is a critical technical advance that will likely be necessary to capture the full diversity of cell types in many brain regions. Patch-seq should be compatible with direct morphological recovery since the axonal and dendritic arbours, which comprise the majority of morphological variability, remain physically embedded in the tissue slice even if the cell body is distorted or damaged during aspiration of cell contents into the patch pipette. We have made two key modifications to our original internal solution recipe to enable direct morphological recovery of Patch-seq neurons. First, we added biocytin into the internal solution, which is the standard dye used in electrophysiology to fill cells and later visualize their morphology. Second, we brought the osmolarity into a more physiological range to allow longer recordings, giving sufficient time for biocytin to diffuse throughout the fine cellular processes. Using this modified internal solution we have been able to recover morphology directly from Patch-seq neurons (Fig. 2).

## Your Patch-seq protocol is based on the Smart-seq2 sequencing method. Can it be combined with other sequencing methods?

Our basic protocol for isolating single-cell RNA could potentially be combined with any plate-based library preparation and sequencing method, but would not be compatible with droplet-based or microfluidic cell-sorting sequencing technologies due to the need to patch the cell in situ in order to collect meaningful physiological measurements. As mentioned above, the Smart-seq2 sequencing method that we based our protocol on has been demonstrated to have excellent sensitivity and full-length coverage in a wide range of cell types and experimental settings [11, 14, 15]. However, sequencing technology is continually evolving and it is inevitable that the Patch-seq protocol may need to be updated to keep pace with these advancements. An important consideration when choosing a sequencing method, particularly for Patch-seq studies that tend to have lower throughput, is the importance of being able to compare the results with the previous literature. Each sequencing method can be prone to unique biases, making it difficult to compare results from one study to another simply because of differences in sequencing methods. An added benefit of using Smart-seq2 has been that we can compare our scRNA-seq data with reference databases generated from the Allen Institute of Brain Science, which is also using a Smart-seq2-based sequencing approach to generate large data sets from dissociated neurons [4].

## How is Patch-seq different from previous techniques that used qRT-PCR or microarray following patch-clamp recording?

Many groups have attempted to combine whole-cell patch clamp recording with analysis of single-cell gene expression. Early studies performed RT-PCR of the patch pipette contents following whole-cell recording [7–9]. However, these techniques require the prior selection of a handful of genes (up to a few dozen [16, 17]), providing only a small glimpse of the full complexity of genes expressed by each cell and precluding the identification of novel genes that may be important in determining cellular phenotype. Single-cell microarray has also been used to assess a larger number of genes following patch clamp recording [18]; however, microarray techniques have a limited dynamic range and poor sensitivity and specificity compared to sequencing-based approaches [19, 20], and cannot detect novel transcripts or splice variants. One previous attempt to perform scRNA-seq on patched neurons yielded relatively poor quality sequencing data (compared to scRNA-seq of dissociated neurons): three neurons from acute brain slices were sequenced with an average of <2000 genes detected per cell and a mean correlation of ~0.25 across cells, suggesting high technical variability between samples [21]. In contrast, our protocol yields high quality scRNA-seq data comparable to that obtained from dissociated cells [3, 4], with a mean correlation of ~0.6 and ~7000 genes detected per cell [1]. Thus, our Patch-seq technique represents a substantial advance in the ability to profile genome-wide expression patterns of patch clamp recorded neurons.

## What are the most critical steps of the Patch-seq protocol?

Strict RNase-free preparation of solutions and handling of samples is absolutely critical to the success of the experiment. RNase is ubiquitous in the environment [22, 23] and a small amount of RNase contamination prior to reverse transcription can easily degrade all of the RNA contained in a single cell (estimated to be ~10 pg). In addition to RNase-free precautions, the quality of the tissue slice, cell, and the patching steps themselves are all important to be able to isolate high-quality RNA from single cells. If the cell is unhealthy, it will be difficult to patch and the RNA may be degraded already by the time it is collected. Likewise, if the recording is unstable or there is significant current leakage, RNases from the extracellular space may enter the pipette and degrade the RNA. In our experience, the best cDNA yield comes from cells that are healthy, were patched quickly, and had stable recordings requiring minimal holding currents. Under these conditions the cell can typically tolerate a significant amount of negative pressure to aspirate the cell cytoplasm and organelles into the patch pipette. The aspiration process should be tailored to each individual cell since cell size and shape, pipette tip size, angle of approach, and many other factors can affect the aspiration process. Since expertise in both molecular biology and electrophysiology are equally critical to success, Patch-seq experiments are well suited to collaborations between two or more individuals with complementary skill sets.

## Are there any special challenges to consider when attempting Patch-seq in an intact animal?

**Fig. 3.**
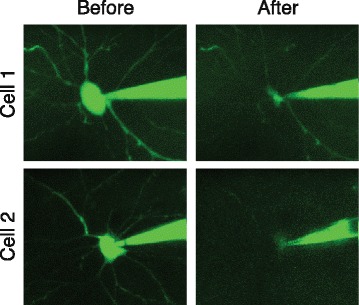
Collapse of cell body during aspiration into patch pipette. Two example neurons patched in vivo under two-photon guidance using a green fluorescent dye in the patch pipette. In both cases, the cell body was noted to decrease in size dramatically after aspirating the cell contents into the pipette. Adapted by permission from Macmillan Publishers Ltd: *Nature Biotechnology* [1], copyright (2016)

Patching in vivo is generally more difficult than patching in slices, and Patch-seq experiments are no exception. In addition to the difficulty of patching itself, the pipette has to travel a further distance through tissue to reach the target cell and may pick up debris from the extracellular space. It is therefore especially important to maintain positive pressure while advancing the pipette through the tissue. The dura should be removed so that it does not stick to the pipette. A fluorescent dye can be used to visualize the pipette tip and surrounding cells (which appear as shadows compared to the fluorescence diffusing into the extracellular space) using two-photon imaging, and to monitor the cell body of the patched neuron during aspiration (Fig. 3) since differential interference contrast (DIC) microscopy is not an option in vivo. The electrical properties of the cell, including its firing pattern, may also be more difficult to quantify in vivo due to more ongoing activity within the intact circuit, and so the recording session should be thoughtfully designed to focus on high priority aspects of the cell’s physiology during the recording session. While undoubtedly more challenging, in vivo Patch-seq experiments have the potential to address important questions that cannot be studied in slices, such as investigating the relationship between the tuning properties of neurons and their transcriptome. In addition, some cell types have long-range axons and elaborate dendritic arbors that may not survive the slicing procedure and therefore need to be targeted in the intact animal.

## Are there any physiologic signs I can look for to determine whether the cell contents have entered the pipette?

When studying a deep brain structure, one may not have the luxury of visualizing the cell body during the experiment. In such cases, it may be possible to indirectly assess whether cell contents have entered the pipette by carefully monitoring the access resistance. We often observe a steady increase in access resistance that seems to correlate with entry of cell contents (especially large organelles such as the nucleus) into the patch pipette. When the organelles completely pass the tip of the pipette there may be a steep decrease in resistance and very little, if any, suction is needed to aspirate the remaining cell cytoplasm. While we typically use visual guidance to confirm that the cell body has collapsed, it may be possible to use these electrophysiological signs as a proxy for determining when the cell contents have been successfully aspirated. As mentioned above, a fluorescent dye can also be added to the internal solution to visualize cells up to several hundred microns deep using multi-photon imaging in vivo.

## What is the throughput of this technique, and what are the limiting factors?

In our lab, with two to three people working together on a single setup, we can collect 30–40 Patch-seq samples per day. If morphological recovery is also required, then only 10–15 samples are collected per day due to the longer recording time required to allow biocytin to diffuse into the fine cellular processes. The main limiting factor to maximizing throughput is the skill and speed of the electrophysiologist(s). Patch clamp recording is a high-level skill that can take years to master. Ultimately, automation of the patching procedure may further increase throughput and reduce the intensive human effort required to collect Patch-seq samples [24]. Improvements to the internal solution and/or staining procedures that can reduce the recording time required for morphological recovery could also potentially increase the throughput of combined sequencing and morphological analysis of neurons.

## What is the average cost per cell?

By using off-the-shelf and in-house-produced reagents whenever possible we have reduced the cost of generating single-cell cDNA libraries to approximately $21/cell. This excludes upfront capital expenditures for equipment (electrophysiology rig, Agilent Bioanalyzer, and basic lab supplies) as well as sequencing costs, which vary widely depending on the sequencing depth and institutional core resources.

## I am an electrophysiologist. How difficult would it be to implement the Patch-seq technique in my lab?

An electrophysiologist would already have the vast majority of equipment and expertise needed to begin Patch-seq experiments in his or her lab. A few additional pieces of equipment may need to be purchased, such as a biosafety cabinet, Bioanalyzer, and Qubit fluorometer. The main challenges likely to be faced involve adopting the RNase-free habits necessary for working with picogram quantities of RNA including infallible sterile technique and a healthy degree of RNase paranoia, as these skills are not typically required for electrophysiology experiments. It could be beneficial, at least initially, to work with a molecular biologist experienced in single-cell RNA work until these habits become second nature.

## I am a molecular biologist. How difficult would it be to implement the Patch-seq technique in my lab?

Patch clamping requires specialized equipment and expertise that may be difficult to pick up quickly or cheaply. In our experience, each step of the patching process—the quality of the tissue slices, the efficiency of patching a neuron, the stability of the recording, and the interactive process of aspirating cell contents—is critical to obtain high quality RNA from single cells. The ability to quickly and intuitively understand how a cell ‘is doing’ typically emerges as a result of experience recording from many neurons. It may not be time- or cost-effective for a molecular biologist to embark on acquiring these skills themselves if there is a potential collaborator available who has already mastered the technique. This is not to say it is impossible for a molecular biologist to do these experiments on his or her own, only that he or she should be prepared to devote sufficient time and resources to achieve proficiency.

## Why am I getting very little, if any, full-length cDNA?

The problem can be localized to one (or both) of two experimental stages: sample processing or sample collection. To determine whether there is a problem during sample processing, we recommend first optimizing the entire protocol using a known total RNA spike-in of ~10 pg to ensure that if there is sample content present, it is not being lost along the way due to RNase contamination, inefficient reverse transcription, poor amplification, and other steps. The positive control RNA spike-in can either be purchased from commercial sources or isolated from whole brain and diluted to approximate the amount in a single neuron (~10–30 pg). If this small quantity of known input RNA does not consistently yield high quality full-length cDNA, there is no hope that a patched neuron’s RNA will give any better results and the protocol should be further optimized before attempting to amplify cDNA from patched neurons. Problems during sample processing must be addressed before considering problems during sample collection.

If high-quality, full-length cDNA can routinely be generated from ~10 pg known RNA input, but not from patched neurons, then the problem may be during sample collection. The most common issues we have come across during this phase of the experiment are: 1) the sample is collected into too much internal solution, which acts as a potent inhibitor of reverse transcription [1]; 2) the internal solution is contaminated with RNase and needs to be re-made; 3) the cell and/or tissue slice was unhealthy, or the patch was poor quality, and the RNA was already degraded by the time the sample was collected; 4) insufficient suction was used to aspirate cell contents into the patch pipette, which may be secondary to the cell being unstable and unable to tolerate suction; or 5) too much suction resulted in aspiration of extracellular material including RNases into the patch pipette.

Even after the Patch-seq technique is well established in the lab, it is critical to continue using appropriate positive and negative controls for each experiment to detect and localize problems (such as new contamination of a solution with RNase or previously amplified cDNA) as soon as they arise.

## What alternative strategies exist to link morphology, electrophysiology, and transcriptome data?

An alternative approach to Patch-seq is to utilize high-throughput scRNA-seq of dissociated neurons to identify molecularly specified cell types, and then further characterize the morphology and physiology of these molecular cell types using transgenic mouse lines (for example, Cre driver lines). By expressing a fluorescent reporter in the cell type of interest, standard patch-clamp or other techniques can be applied to characterize the physiology and morphology of the targeted cell population. In practice, this approach is often limited by the availability and specificity of driver lines, which frequently label multiple functionally distinct cell types [6]. In addition, this approach assumes transcriptomic and phenotypic homogeneity within the target population and forfeits the ability to resolve additional cell subtypes by dissociating gene expression from phenotype at the level of single cells. The continued development of intersectional genetic tools may enable more specific targeting of molecular subtypes and expand the utility of transgenic approaches to link transcriptomic cell types with their corresponding morphological and electrophysiological phenotypes.
